# Supplementary material for: Does pre-emptive dexamethasone provide prophylaxis against sugammadex-induced bradycardia? A retrospective study
Source: PLoS One. 2025 May 20;20(5):e0323419. doi: 10.1371/journal.pone.0323419 (PMC12091743; doi:10.1371/journal.pone.0323419)
Supplement: Attachment — Submitted filename: Manuscript Review .docx [file pone.0323419.s002.docx]

**Does Pre-emptive Dexamethasone Provide Prophylaxis Against Sugammadex-**

**Induced Bradycardia? A Retrospective Study**

**Introduction**

- Clarification of the hypothesis: The paragraph discussing the hypothesis could be clearer. Specifically, the idea of a “mini Addisonian crisis” due to corticosteroid binding and unbinding may need further clarification. A bit more background on Addisonian crisis and its pathophysiology could help the reader understand why this phenomenon is relevant here.

Suggested revision: “Jahr and colleagues hypothesized that sugammadex-induced bradycardia may be due to a transient decrease in circulating corticosteroids, causing a temporary ‘mini Addisonian crisis.’ This is thought to resolve when sugammadex preferentially binds to neuromuscular blockers, thus reducing corticosteroid binding in the bloodstream.”

**Methods**

- Clarification of "prospective study" reference: In the second sentence, you mentioned a “prospective study on sugammadex-related bradycardia on November 29, 2022.” It might be helpful to clarify what this study is and how it relates to the current retrospective analysis. Is this an ongoing study, or was it completed by the time the retrospective analysis was performed?

- Statistical details: You have outlined the statistical methods well, but the power and sample size calculation could be more clearly placed before the results to avoid repetition in the discussion section.

**Results**

- Clarify the findings: In the results section, it is mentioned that there was no statistically significant difference between the groups for average heart rate change (p = 0.55) and maximal heart rate change (p = 0.23). You might want to emphasize that while there were differences in the heart rate changes between the groups, these differences were not statistically significant. This could help guide the reader's expectations regarding the impact of dexamethasone.

Suggested revision: "Although the dexamethasone group showed a slightly greater maximal heart rate slowing (2.1 bpm vs. 0.9 bpm in the control group), this difference was not statistically significant (p = 0.23)."

- Clarification on beta-blocker use: You mention that the dexamethasone group had fewer patients on beta-blockers, but it's not clear how this might affect the interpretation of results. More explanation of why this difference matters and how you adjusted for it in your analysis would strengthen the interpretation.

**Discussion**

- Discussion of corticosteroids: While you mention that hydrocortisone or fludrocortisone might be needed to prevent sugammadex-induced bradycardia, this hypothesis needs further explanation. It’s a good idea to expand on why these particular corticosteroids might be more relevant than dexamethasone (e.g., differences in mineralocorticoid activity).

- Clinical relevance of bradycardia: While you report the frequency of bradycardia in patients on and off beta-blockers, it would be helpful to discuss the clinical significance of these bradycardic events. Even though no significant effects were found, could the bradycardia still be clinically relevant, especially in the perioperative setting?

- Beta-blocker use and sugammadex: You discuss the association between beta-blocker use and sugammadex-induced bradycardia but stop short of drawing any firm conclusions. This is understandable given the statistical results, but offering some guidance or suggestions for future studies could help. A recommendation for larger studies or prospective trials to evaluate the interaction between beta-blockers and sugammadex would be valuable.

**General Comments**

- Grammar and clarity: The manuscript is generally well-written, but there are several places where sentences could be restructured for greater clarity and readability. Minor grammatical corrections are needed, such as in verb tense consistency and article usage.

- Consistency in terminology**: Make sure to be consistent when referring to drug names and medical terms throughout the manuscript (e.g., "sugammadex" vs "Sugammadex").

**Final Thoughts:**

Overall, the manuscript presents an interesting and relevant study. The topic of sugammadex-induced bradycardia and its interaction with dexamethasone and beta-blockers is important, but the findings in this study suggest that the hypotheses might need further refinement. The suggestions provided above will help improve clarity, precision, and overall readability. Good work, and I encourage further exploration of this topic!
