# Supplementary material for: Does pre-emptive dexamethasone provide prophylaxis against sugammadex-induced bradycardia? A retrospective study
Source: PLoS One. 2025 May 20;20(5):e0323419. doi: 10.1371/journal.pone.0323419 (PMC12091743; doi:10.1371/journal.pone.0323419)
Supplement: Attachment — Submitted filename: Response to Reviewers.docx [file pone.0323419.s003.docx]

Response to Reviewers

Manuscript: PONE-D-24-42641

Does Pre-emptive Dexamethasone Provide Prophylaxis Against Sugammadex-Induced Bradycardia? A Retrospective Study

The authors appreciate the Academic Editor’s and Reviewers’ helpful comments, which we have addressed below. Academic Editor and Reviewer comments are provided below in italics, with our subsequent response in blue.

**Academic Editor:**

*1. Please ensure that your manuscript meets PLOS ONE's style requirements, including those for file naming. The PLOS ONE style templates can be found at*

*https://journals.plos.org/plosone/s/file?id=wjVg/PLOSOne_formatting_sample_main_body.pdf and*

*https://journals.plos.org/plosone/s/file?id=ba62/PLOSOne_formatting_sample_title_authors_affiliations.pdf*

**Response:** We thank you for this guidance and have updated the manuscript to meet PLOS ONE’s style requirements, including those for file naming.

*2. When completing the data availability statement of the submission form, you indicated that you will make your data available on acceptance. We strongly recommend all authors decide on a data sharing plan before acceptance, as the process can be lengthy and hold up publication timelines. Please note that, though access restrictions are acceptable now, your entire data will need to be made freely accessible if your manuscript is accepted for publication. This policy applies to all data except where public deposition would breach compliance with the protocol approved by your research ethics board. If you are unable to adhere to our open data policy, please kindly revise your statement to explain your reasoning and we will seek the editor's input on an exemption. Please be assured that, once you have provided your new statement, the assessment of your exemption will not hold up the peer review process.*

**Response:** We thank you for this recommendation and will make our data freely accessible if accepted for publication. The authors agree to place the data under Supplemental Information as “S1 File”.

*3. Please amend either the abstract on the online submission form (via Edit Submission) or the abstract in the manuscript so that they are identical.*

**Response:** We thank the editor for bringing this to our attention. We have amended the abstract on the online submission form to match the abstract in the manuscript.

**Reviewers:**

*1. Is the manuscript technically sound, and do the data support the conclusions?

The manuscript must describe a technically sound piece of scientific research with data that supports the conclusions. Experiments must have been conducted rigorously, with appropriate controls, replication, and sample sizes. The conclusions must be drawn appropriately based on the data presented.*

*Reviewer #1: Partly*

**Response:** We thank the reviewer for this comment. We sincerely believe that the data presented in this manuscript is derived from rigorously conducted experiments. The study's dataset originates from a prospective investigation into sugammadex-related bradycardia (PMID: 35759402, DOI: 10.1213/ANE.0000000000006131), which was published in Anesthesia & Analgesia in 2022.

*Reviewer #2: No*

**Response:** Thank you for this comment. This retrospective study was conducted to explore a published hypothesis regarding a potential mechanism for sugammadex-related bradycardia (PMID: 36608072, DOI: 10.1097/MJT.0000000000001590). Although the results did not support the hypothesis, we believe this study is important to share because sugammadex is widely administered during anesthesia, and the cause of sugammadex-induced bradycardia remains unknown, with possibly clinically significant implications.

*2. Has the statistical analysis been performed appropriately and rigorously?*

*Reviewer #1: Yes*

**Response:** We thank the reviewer for this positive comment.

*Reviewer #2: I Don't Know*

**Response:** We thank the reviewer for this comment. We appreciate your inquiry regarding the appropriateness and rigor of the statistical analysis. The statistical methods used in this study were carefully selected and conducted by a biostatistician with over 10 years of experience (Master’s-level training). The analyses were performed in accordance with standard statistical practices and guidelines, ensuring robust and reliable results.

*3. Have the authors made all data underlying the findings in their manuscript fully available?

The* [*PLOS Data policy*](https://urldefense.com/v3/__http:/track.editorialmanager.com/CL0/http:*2F*2Fwww.plosone.org*2Fstatic*2Fpolicies.action*23sharing/1/010f0193b8447352-d0004011-6917-4848-9853-40247b745178-000000/k-H7ChZJlgIiNPyS8v1VFx053bQ3QY9NHYu9nMdP3bQ=189__;JSUlJSU!!F9wkZZsI-LA!FJm_MRXYrQkIbawwLWFDX0zAV11_SaINSYEl9Kige5FaNHWDopZNQjj33_JFCs14AvSXcbkdRQJf8NK-1csS7hw$) *requires authors to make all data underlying the findings described in their manuscript fully available without restriction, with rare exception (please refer to the Data Availability Statement in the manuscript PDF file). The data should be provided as part of the manuscript or its supporting information, or deposited to a public repository. For example, in addition to summary statistics, the data points behind means, medians and variance measures should be available. If there are restrictions on publicly sharing data—e.g. participant privacy or use of data from a third party—those must be specified.*

*Reviewer #1: Yes*

**Response:** We thank the reviewer for this positive comment.

*Reviewer #2: Yes*

**Response:** We thank the reviewer for this positive comment.

*4. Is the manuscript presented in an intelligible fashion and written in standard English?

PLOS ONE does not copyedit accepted manuscripts, so the language in submitted articles must be clear, correct, and unambiguous. Any typographical or grammatical errors should be corrected at revision, so please note any specific errors here.*

*Reviewer #1: Yes*

**Response:** We thank the reviewer for this positive comment.

*Reviewer #2: Yes*

**Response:** We thank the reviewer for this positive comment.

*5. Review Comments to the Author

Please use the space provided to explain your answers to the questions above. You may also include additional comments for the author, including concerns about dual publication, research ethics, or publication ethics. (Please upload your review as an attachment if it exceeds 20,000 characters)*

*Reviewer #1: Please find attached the review comments to the author

Overall, the manuscript presents an interesting and relevant study. The topic of sugammadex-induced bradycardia and its interaction with dexamethasone and beta-blockers is important, but the findings in this study suggest that the hypotheses might need further refinement. The suggestions provided above will help improve clarity, precision, and overall readability. Good work, and I encourage further exploration of this topic!*

**Response: We thank the reviewer for the positive feedback. We believe this study is important to share because sugammadex is widely administered during anesthesia, yet the cause of sugammadex-induced bradycardia remains unknown. Our aim is to address this gap by testing our hypothesis through this retrospective analysis. Publishing this report will help disseminate current knowledge, encourage other groups to explore the question prospectively, and investigate potential relationships.**

*Reviewer #2: 1. It is unclear how the sample size was calculated. It seems like it just reflects the number of patients that were found on the database rather than the number of participants required to detect a difference in the results. I would recommend a statistician review the sample size calculation and the statistical analyses. Please confirm the primary outcome and whether the sample size was powered on this.*

**Response:** The sample size for this study was determined based on logistical constraints, specifically the time period during which two medical students (VBK and MTA) were available to assist with data collection (May to September 2021). While the sample size was not chosen prospectively based on a formal power calculation for a specific primary outcome, we retrospectively computed the effect sizes that could be detected with adequate power (>80%) for the available sample size.

For binary outcomes, this sample size provides adequate power to detect differences as small as 30% (e.g., p1=35%, p2=65%; two-tailed alpha=0.05, group weights 2:1) using a chi-square test. For continuous outcomes, we computed that the sample size could detect effect sizes (Cohen’s d) as small as 0.6 with adequate power (>80%) using a two-sample t-test with the same alpha and group weights.

While the primary outcomes of interest were average HR change and max HR slowing, the retrospective calculation suggests that the study was not adequately powered to detect the smaller observed effect sizes (0.12 for HR change and 0.003 for max HR slowing). As such, the ability to detect significant differences for these specific outcomes was limited by the small observed effect sizes in our dataset. Additional detail about the sample size calculation were added under “Methods” (paragraph 3) and the limitations of our sample size were acknowledged under “Discussion” (paragraph 2).

Methods: Power and sample size calculation

“An a priori sample size calculation was not computed for this study. The sample size was determined based on a 3 month period (May to September 2021) when two medical students (VBK and MTA) were available to assist with data collection. After determining the available sample size, a retrospective analysis computed the effect sizes that could be detected with adequate power (>80%). It was determined that a sample size of 103 provided adequate power (>80%) to detect effect sizes as small as 30% for binary outcomes (e.g. p1=35%, p2=65%, two-tailed alpha=0.05, group weights 2:1 using the chi-square test). For continuous outcomes, this sample size provided adequate power (>80%) to detect effect sizes (Cohen’s d) as small as 0.6 between groups (two-sample t-test, two-tailed alpha=0.05, group weights 2:1).”

Discussion:

“Furthermore, the small sample size was a limitation of the study. The retrospective calculation suggested that this study was not adequately powered to detect the smaller observed effect sizes (0.12 for average HR change and 0.003 for max HR slowing). As such, the ability to detect significant differences for these specific outcomes was limited by the small observed effect sizes in our dataset.”

*2. There is no mention of the dose of dexamethasone that these participants received, or when or how it was administered (beyond the mention of 4-8mg in the Background section and that they had received this prior to sugammedex). Please include further details on this.*

**Response:** We thank the reviewer for the suggestion to expand on the specifics of the dosing and administration of dexamethasone. We reviewed our records and determined the dexamethasone doses administered to the 38 patients in the treatment group. The dose of dexamethasone administered intravenously was either 4 mg, 8 mg, or 10 mg. This information was added under “Results” (paragraph 1). This breakdown of the administered doses was also added to Table 1 and was as follows:

- 4 mg: 9 patients (8.7%)
- 8 mg: 17 patients (16.5%)
- 10 mg: 12 patients (11.7%)

Results:

The study database consisted of 103 subjects, of whom 38 received intravenous dexamethasone (either 4 mg, 8 mg, or 10 mg) during their anesthetic course prior to sugammadex and 65 patients were identified as not receiving dexamethasone.

Table 1:

*3. Please confirm the p value for the change in maximal HR (in the table it is listed as p0.988 but in the text it is 0.23)*

**Response:** We thank the reviewer for the recommended edit for clarity. The p-value for the change in maximal heart rate is 0.998 (Fig 1, Table 2) and 0.907 in the adjusted model. The adjusted model accounts for all variables in Table 1, including race, age, BMI, ASA classification, hypertension, diabetes, coronary artery disease, malignancy, outpatient beta-blocker medications, insulin use, and ACEI/ARBs. The text has been updated to reflect this under Results (paragraph 1).

“The average heart slowing (3.2 bpm ± 3.9 in the control group, 3.7 bpm ± 3.8 in the dexamethasone group), and maximal HR slowing (5.0 bpm ± 3.9 in the control group, 5.0 ± 3.8 in the dexamethasone group) over the five minutes following sugammadex administration was determined in each group (Table 2). Although the dexamethasone group showed a slightly greater average HR slowing (3.7 bpm ± 3.8 vs 3.2 ± 3.9 in the control group), this difference was not statistically significant (p = 0.553, Fig 1). The maximal HR slowing was also not significant between groups (p = 0.988, Fig 1). After adjusting for baseline patient characteristics, the adjusted p-values were 0.570 and 0.907, respectively.”

*4. I am unsure the sample size in this study is large enough to support or negate the original hypothesis, as the authors state in the Discussion.*

**Response:** We thank the reviewer for the comment. The sample size for this study was determined based on logistical constraints, specifically the time period during which two medical students (VBK and MTA) were available to assist with data collection (May to September 2021). While the sample size was not chosen prospectively based on a formal power calculation for a specific primary outcome, we retrospectively computed the effect sizes that could be detected with adequate power (>80%) for the available sample size.

For binary outcomes, this sample size provides adequate power to detect differences as small as 30% (e.g., p1=35%, p2=65%; two-tailed alpha=0.05, group weights 2:1) using a chi-square test. For continuous outcomes, we computed that the sample size could detect effect sizes (Cohen’s d) as small as 0.6 with adequate power (>80%) using a two-sample t-test with the same alpha and group weights.

While the primary outcomes of interest were average HR change and maximal HR slowing, the retrospective calculation suggests that the study was not adequately powered to detect the smaller observed effect sizes (0.12 for HR change and 0.003 for max HR slowing). As such, the ability to detect significant differences for these specific outcomes was limited by the small observed effect sizes in our dataset. Additional detail about the sample size calculation were added under “Methods” (paragraph 3) and the limitations of our sample size were acknowledged under “Discussion” (paragraph 2).

Methods: Power and sample size calculation

“An a priori sample size calculation was not computed for this study. The sample size was determined based on a 3 month period (May to September 2021) when two medical students (VBK and MTA) were available to assist with data collection. After determining the available sample size, a retrospective analysis computed the effect sizes that could be detected with adequate power (>80%). It was determined that a sample size of 103 provided adequate power (>80%) to detect effect sizes as small as 30% for binary outcomes (e.g. p1=35%, p2=65%, two-tailed alpha=0.05, group weights 2:1 using the chi-square test). For continuous outcomes, this sample size provided adequate power (>80%) to detect effect sizes (Cohen’s d) as small as 0.6 between groups (two-sample t-test, two-tailed alpha=0.05, group weights 2:1).”

Discussion:

“Furthermore, the small sample size was a limitation of the study. The retrospective calculation suggested that this study was not adequately powered to detect the smaller observed effect sizes (0.12 for average HR change and 0.003 for max HR slowing). As such, the ability to detect significant differences for these specific outcomes was limited by the small observed effect sizes in our dataset.”

*5. Please include the results of the secondary analyses (i.e. effect of peri-operative beta-blockers on bradycardia) in the Results section. How many participants experienced bradycardia after sugammedex? How was bradycardia defined?*

**Response:** We thank the reviewer for this request**. We evaluated the effect of perioperative beta-blockers (BB) on the incidence of bradycardia (defined as HR less than 60 bpm), given the potential mechanistic role of beta-blockers in modulating heart rate. We performed adjusted analyses to account for beta-blocker usage as well as other covariates listed in Table 1 (**including race, age, BMI, ASA classification, hypertension, diabetes, coronary artery disease, malignancy, insulin use, and ACEI/ARBs**).**

**The findings are as follows:**

- **Overall Cohort**:
  - Without perioperative beta-blockers: 38.3% (31/81) experienced bradycardia.
  - With perioperative beta-blockers: 59.1% (13/22) experienced bradycardia.
  - This difference was not statistically significant (p=0.080, chi-square test)
- **Subgroup Analysis (Patients on beta-blockers)**:
  - No Dexamethasone: 29.8% (14/47) experienced bradycardia.
  - Dexamethasone: 50.0% (17/34) experienced bradycardia.
  - This difference approached statistical significance (p=0.065, chi-square test).

**These findings suggest that beta-blocker usage may influence the incidence of bradycardia, though the results did not reach statistical significance. Adjusted analyses confirmed that beta-blocker usage did not confound the primary results related to heart rate changes, as the adjusted p-values were consistent with the unadjusted analyses. We have included these findings and the definition of bradycardia in the revised manuscript under the “Results” section (paragraph 3).**

“The dexamethasone group had significantly fewer patients on beta blockers as an outpatient medication (10.5% vs 27.7%, p = 0.04) and was adjusted for in our secondary analysis, where the effect of perioperative beta-blocker use was included in the models. We also evaluated the incidence of bradycardia (defined as HR less than 60 bpm). In the overall cohort, 38.3% (31/81) of subjects not on beta-blockers experienced bradycardia compared to 59.1% (13/22) of those on beta-blockers. This difference was not statistically significant (p = 0.080). Among patients on beta-blockers, the subgroup analysis revealed that bradycardia occurred in 29.8% (14/47) of those who did not receive dexamethasone and 50.0% (17/34) of those who did, with this difference approaching statistical significance (p = 0.065). Adjusted analyses accounting for beta-blocker use yielded p-values that were consistent with the unadjusted analyses. **Adjusted analyses confirmed that beta-blocker usage did not confound the primary results related to heart rate changes, as the adjusted p-values were consistent with the unadjusted analyses.**”

**Introduction**

*4. Clarification of the hypothesis: The paragraph discussing the hypothesis could be clearer. Specifically, the idea of a “mini Addisonian crisis” due to corticosteroid binding and unbinding may need further clarification. A bit more background on Addisonian crisis and its pathophysiology could help the reader understand why this phenomenon is relevant here.*

*Suggested revision: “Jahr and colleagues hypothesized that sugammadex-induced bradycardia may be due to a transient decrease in circulating corticosteroids, causing a temporary ‘mini Addisonian crisis.’ This is thought to resolve when sugammadex preferentially binds to neuromuscular blockers, thus reducing corticosteroid binding in the bloodstream.”*

**Response:** We thank the editor and reviewers for the suggested revision to clarify the hypothesis. We have amended the language in the Introduction (paragraph 2). Jahr and colleagues proposed that sugammadex-induced bradycardia may result from its initial binding to corticosteroids, leading to a temporary ‘mini Addisonian crisis’ due to reduced circulating corticosteroid levels. Sugammadex has a high binding affinity for steroidal neuromuscular blocking agents like rocuronium (Ka of 1.79 × 10⁷ mol/L) and vecuronium (Ka of 5.72 × 10⁶ mol/L) but a much weaker affinity for corticosteroids, as shown by the Ka values for dexamethasone (<1.00 × 10³ mol/L), hydrocortisone (5.48 × 10⁴ mol/L), and fludrocortisone (1.00 × 10⁵ mol/L) [6,7]. This transient corticosteroid binding may explain reports of bradycardia or cardiac arrest that are resistant to catecholamines and anticholinergics in predisposed patients. However, as sugammadex moves to peripheral sites like muscle vascular beds, it preferentially binds to neuromuscular blockers, releasing corticosteroids back into circulation and potentially reducing the risk of severe bradycardia.

“Jahr and colleagues hypothesized that sugammadex-induced bradycardia may be due to a transient decrease in circulating corticosteroids, causing a temporary ‘mini Addisonian crisis.’ This is thought to resolve when sugammadex preferentially binds to neuromuscular blockers, thus reducing corticosteroid binding in the bloodstream [4,5]. This theory is based on the binding affinity of sugammadex. Sugammadex has a high binding affinity for steroidal neuromuscular blocking agents, such as rocuronium (Ka of 1.79 x 10^7^ mol/L) and vecuronium (Ka of 5.72 x 10^6^ mol/L) [6,7]. Comparatively, the binding affinity to corticosteroids is relatively weak, as indicated by the Ka values: dexamethasone (<1.00 x 10^3^ mol/L), hydrocortisone (5.48 x 10^4^ mol/L), and fludrocortisone (1.00 x 10^5^ mol/L) [6,7]. When administered, sugammadex may initially bind to steroids in the central circulation. In patients predisposed to bradycardia due to reduced corticosteroid concentrations, this initial binding could theoretically contribute to the effect, and may explain reports of cardiac arrest refractory to catecholamines and anticholinergics. However, as sugammadex circulates to peripheral sites, including muscle vascular beds, it would preferentially bind to rocuronium or vecuronium. This binding would subsequently release corticosteroids back into the circulation, potentially reducing the risk of more severe bradycardia.”

***Methods***

*5. Clarification of "prospective study" reference: In the second sentence, you mentioned a “prospective study on sugammadex-related bradycardia on November 29, 2022.” It might be helpful to clarify what this study is and how it relates to the current retrospective analysis. Is this an ongoing study, or was it completed by the time the retrospective analysis was performed?*

**Response:** We thank the editor for the suggested clarification. The prospective study, published in Anesthesia & Analgesia in 2022, investigated heart rate (HR) responses to sugammadex reversal. Importantly, the study demonstrated that sugammadex, when used for muscle relaxant reversal, can rapidly decrease HR, although the degree of HR reduction varied. This well-designed and published study also concluded that the mechanism underlying this HR slowing remains unknown. These findings prompted us to retrospectively analyze the same dataset to evaluate our hypothesis regarding sugammadex-related bradycardia. We have included additional details about the original study and the relevance of the cohort used for our retrospective analysis in the “Methods” section (paragraph 1).

“The prospective study investigated HR responses to sugammadex antagonism. Importantly, the study demonstrated that sugammadex, when used for muscle relaxant reversal, may rapidly decrease HR, although the degree of HR reduction varied. The same published data set was used for the retrospective analysis.”

*6. Statistical details: You have outlined the statistical methods well, but the power and sample size calculation could be more clearly placed before the results to avoid repetition in the discussion section.*

**Response:** We thank the editor and reviewers for this suggestion and agree with the recommended placement of the power and sample size calculation.

***Results***

*7. Clarify the findings: In the results section, it is mentioned that there was no statistically significant difference between the groups for average heart rate change (p = 0.55) and maximal heart rate change (p = 0.23). You might want to emphasize that while there were differences in the heart rate changes between the groups, these differences were not statistically significant. This could help guide the reader's expectations regarding the impact of dexamethasone.*

*Suggested revision: "Although the dexamethasone group showed a slightly greater maximal heart rate slowing (2.1 bpm vs. 0.9 bpm in the control group), this difference was not statistically significant (p = 0.23)."*

**Response:** We thank the editor and reviewers for this excellent suggestion. We have made the recommended edit under “Results” (paragraph 1)

“Although the dexamethasone group showed a slightly greater average HR slowing (3.7 bpm ± 3.8 vs 3.2 ± 3.9 in the control group), this difference was not statistically significant (p = 0.553, Fig 1). The maximal HR slowing was also not significant between groups (p = 0.988, Fig 1). After adjusting for baseline patient characteristics, the adjusted p-values were 0.570 and 0.907, respectively.”

*8. Clarification on beta-blocker use: You mention that the dexamethasone group had fewer patients on beta-blockers, but it's not clear how this might affect the interpretation of results. More explanation of why this difference matters and how you adjusted for it in your analysis would strengthen the interpretation.*

**Response:** We thank the editor reviewers for highlighting this point. In our analysis, the dexamethasone and control groups were comparable in terms of demographics and comorbidities, except for outpatient beta-blocker use. Beta-blockers are known to intentionally slow heart rate, making it essential to account for their use when evaluating whether dexamethasone attenuates bradycardia.

To address this, we included beta-blocker use and other variables (race, age, BMI, ASA classification, hypertension, diabetes, coronary artery disease, malignancy, beta-blocker use, insulin use, and ACEI/ARBs) as covariates in our adjusted analyses of heart rate outcomes (average HR change and maximal HR slowing). By doing so, we ensured that any observed effects on bradycardia or outcomes on heart rate could be attributed to dexamethasone or sugammadex, rather than confounding patient-related factors, such as perioperative beta-blocker use. I**mportantly, the adjusted analyses yielded p-values that were consistent with the unadjusted analyses, with no change in the conclusions.** This adjustment strengthens the interpretation of our findings and provides a more robust analysis of the potential mechanisms underlying the observed effects. We described this under “Results” (paragraph 3).

“The dexamethasone group had significantly fewer patients on beta blockers as an outpatient medication (10.5% vs 27.7%, p = 0.04) and was adjusted for in our secondary analysis, where the effect of perioperative beta-blocker use was included in the models. We also evaluated the incidence of bradycardia (defined as HR less than 60 bpm). In the overall cohort, 38.3% (31/81) of subjects not on beta-blockers experienced bradycardia compared to 59.1% (13/22) of those on beta-blockers. This difference was not statistically significant (p = 0.080). Among patients on beta-blockers, the subgroup analysis revealed that bradycardia occurred in 29.8% (14/47) of those who did not receive dexamethasone and 50.0% (17/34) of those who did, with this difference approaching statistical significance (p = 0.065). Adjusted analyses accounting for beta-blocker use yielded p-values that were consistent with the unadjusted analyses. **Adjusted analyses confirmed that beta-blocker usage did not confound the primary results related to heart rate changes, as the adjusted p-values were consistent with the unadjusted analyses.”**

***Discussion***

*9. Discussion of corticosteroids: While you mention that hydrocortisone or fludrocortisone might be needed to prevent sugammadex-induced bradycardia, this hypothesis needs further explanation. It’s a good idea to expand on why these particular corticosteroids might be more relevant than dexamethasone (e.g., differences in mineralocorticoid activity).*

**Response:** We thank the editor and reviewers for this excellent suggestion. Dexamethasone is a synthetic corticosteroid with negligeable mineralocorticoid activity. In comparison, fludrocortisone has potent mineralocorticoid activity, while hydrocortisone exhibits both mineralocorticoid and glucocorticoid activity. This information has been added to the “Discussion” (paragraph 1)

“The lack of effect observed with dexamethasone may either negate the theory proposed by Jahr and colleagues [4,5], or it may also be that other corticosteroids, with more mineralocorticoid activity, such as hydrocortisone or fludrocortisone, are required to prevent this effect [10]. Dexamethasone, notably only has glucocorticoid activity and negligible mineralocorticoid activity. In comparison, fludrocortisone has potent mineralocorticoid activity, and hydrocortisone, equal amounts of mineralocorticoid and glucocorticoid activity [10].”

*10. Clinical relevance of bradycardia: While you report the frequency of bradycardia in patients on and off beta-blockers, it would be helpful to discuss the clinical significance of these bradycardic events. Even though no significant effects were found, could the bradycardia still be clinically relevant, especially in the perioperative setting?*

**Response:** We thank the editor and reviewers for this comment. Bradycardia can lead to hypotension, particularly in the context of relative hypovolemia. It may also cause ventricular stretching and increase oxygen consumption due to the work required for contraction. Though it is beyond the scope of this manuscript to discuss the clinical relevance of the observed bradycardic events, it is important to highlight their potential occurrence. As the use of sugammadex becomes increasingly widespread, the likelihood of such bradycardic events may also increase. We have included the potential clinical implications of bradycardia under “Discussion” (paragraph 2).

“This has clinical implications as bradycardia can lead to hypotension, particularly in the context of relative hypovolemia. It may also cause ventricular stretching and increase oxygen consumption due to the work required for contraction.”

*11. Beta-blocker use and sugammadex: You discuss the association between beta-blocker use and sugammadex-induced bradycardia but stop short of drawing any firm conclusions. This is understandable given the statistical results, but offering some guidance or suggestions for future studies could help. A recommendation for larger studies or prospective trials to evaluate the interaction between beta-blockers and sugammadex would be valuable.*

**Response:** We thank the editor and reviewers for this comment. As mentioned, we are unable to make any firm conclusions given the statistical results, but we agree we should provide some guidance for future studies. A clearer recommendation that studies with larger sample sizes or prospective trials would be valuable for future study in this area has been added under “Discussion” (paragraph 2).

"Thus the authors encourage a prospective evaluation with a larger sample size to gain insights in the potential interactions between perioperative beta blocker use and sugammadex.”

***General Comments***

*12. Grammar and clarity: The manuscript is generally well-written, but there are several places where sentences could be restructured for greater clarity and readability. Minor grammatical corrections are needed, such as in verb tense consistency and article usage.*

**Response:** We thank the editor and reviewers for this suggestion. We have reviewed the manuscript for grammar and clarity to improve readability.

*13. Consistency in terminology**: Make sure to be consistent when referring to drug names and medical terms throughout the manuscript (e.g., "sugammadex" vs "Sugammadex").*

**Response:** We thank the editor and reviewers for this suggestion. We have reviewed the manuscript to be consistent when referring to drug names and medical terms throughout the manuscript to improve readability.

***Final Thoughts:***

***14.*** *Overall, the manuscript presents an interesting and relevant study. The topic of sugammadex-induced bradycardia and its interaction with dexamethasone and beta-blockers is important, but the findings in this study suggest that the hypotheses might need further refinement. The suggestions provided above will help improve clarity, precision, and overall readability. Good work, and I encourage further exploration of this topic!*

**Response:** We thank the editor and reviewers for these positive comments.
